# Evolution of the Concept of Sepsis

**DOI:** 10.3390/antibiotics11111581

**Published:** 2022-11-09

**Authors:** Jean-Louis Vincent

**Affiliations:** Department of Intensive Care, Erasme Hospital, Université Libre de Bruxelles, 1070 Brussels, Belgium; jlvincent@intensive.org

**Keywords:** organ dysfunction, inflammation, definition, infection

## Abstract

Sepsis has been recognized for more than 2500 years, but the criteria used to identify it have evolved. Sepsis is an infection associated with some degree of organ dysfunction—put very simplistically, sepsis is a ‘bad infection’. Specific criteria may be useful for research purposes but have less value in day-to-day clinical practice. What is relevant here is early recognition and some awareness of severity so that appropriate therapy can be started without delay.

## 1. Introduction

The first recorded use of the term ‘sepsis’ in a medical sense is found in Greek writings by Homer from more than 2500 years ago, derived from the Greek word σήψη meaning ‘putrefaction’ [1]. The word was also used by Hippocrates, Aristotle, Plutarch and Galen, among others, with a similar meaning of decay or decomposition. These ancient Greek physicians knew little about infectious processes, but recognized sepsis as a pathway to death.

Since those early days, we have become much more knowledgeable about the causes and features of sepsis and several attempts have been made to develop a clear definition of this disease process. Importantly, however, ‘sepsis’ is only a construct that we use to define a situation that can be associated with a number of criteria (or characteristics) and we should not confuse the definition per se with the criteria. Moreover, when speaking about sepsis, we should not talk about diagnosis, which can be defined as the determination of a specific disease entity. Reaching a diagnosis can be reassuring for the patient and the family because it usually precedes a therapeutic plan (which will hopefully treat the disease). As a construct or syndrome, sepsis cannot be ‘diagnosed’ but is ‘recognized’ or ‘identified’. It would not be reassuring for a patient or the family to hear that a ‘diagnosis’ of sepsis has been made if the underlying cause of the sepsis has not been determined.

Other such constructs in medicine include the acute respiratory distress syndrome (ARDS) and entities such as coma or dementia (Table 1).

## 2. Evolution in Concepts: Impact on Definitions

Having a common, uniform definition of any disease process is important, but for day-to-day communication and clinical practice, a really precise definition with strict criteria may not be so relevant; do we find it so difficult to define, for example, pneumonia or myocardial infarction at the bedside (Table 2)? Strict criteria for definitions are more important for research purposes.

### 2.1. The Sepsis Syndrome

The late Roger Bone was the instigator of the first attempt to define sepsis using specific criteria. He introduced the term ‘sepsis syndrome’ in the late 1980s to encompass clinical signs of infection and the presence of inadequate perfusion or dysfunction of at least one end-organ [2]. ‘Sepsis syndrome’ was used in a number of clinical trials on new therapeutic interventions in sepsis (e.g., [3,4]), with few, if any, positive results.

### 2.2. ‘First’ Sepsis Definitions Conference and SIRS

In 1991, a consensus conference led by Roger Bone and organized by the Society of Critical Care Medicine (SCCM) with the American College of Chest Physicians (ACCP) proposed to call sepsis a systemic response to infection. Importantly, at that time, the pathophysiology of sepsis was considered to be essentially a pro-inflammatory state, which could become disproportionate. However, this group of experts made the mistake of considering sepsis as simply a host response characterizing infection, leading to a confusion between the conditions of ‘sepsis’ and ‘infection’. They introduced the construct of ‘systemic inflammatory response syndrome‘ (SIRS) [5], which was based on very simple criteria, i.e., minor alterations in at least two of four simple variables: temperature, heart rate, respiratory rate, and white blood cell (WBC) count. Patients meeting the SIRS criteria who had a presumed or documented infection were considered to have sepsis [6]. The idea was to have a very sensitive means of selecting a broad patient population in order not to miss any patient needing attention.

This consensus conference had considerable impact, both for clinicians and in terms of research, partly because it gathered a large number of eminent North American experts, but largely because the suggested approach was really simple to apply. A key problem with the SIRS-based criteria was that patients with infection (in contrast to colonization) already have signs of systemic response, including, most typically, fever and altered WBC count. Hence, the vast majority of patients with infection can meet the SIRS criteria for sepsis. However, not all patients with infection need urgent attention, so that it is essential to assess the severity of the disease. Sepsis includes some degree of associated organ dysfunction. Although the consensus group proposed that the word ‘severe’ should be added when there was associated organ failure [5], this ‘severe sepsis’ category actually represented patients with sepsis.

### 2.3. ‘Second’ Sepsis Definitions Conference

To address these problems with the SIRS concept, a second consensus conference in 2001 endorsed by a larger group of scientific societies, including some European representation, attempted to reverse the tendency to use the SIRS criteria in the identification of infection and sepsis [7]. The idea was to express the complexity of sepsis and its numerous facets. The participants also wanted to reflect the increase in inflammatory markers, such as C-reactive protein (CRP) and procalcitonin (PCT), in sepsis, in addition to the WBC count, which is not very specific for sepsis.

Table 3 summarizes the list of acute signs, symptoms and abnormalities that were proposed as being associated with sepsis by the participants. Our error (and one that I completely assume as I was responsible for the group of experts making the Table) was that we did not propose some very simple criteria, but rather just a long list of possible signs which may be associated with sepsis.

Unfortunately, largely because SIRS appeared so much simpler, this 2001 definition did not gather much support from physicians or researchers and the SIRS criteria continued to be widely used.

Since patients with ‘infection’ and ‘sepsis’ basically had the same criteria using the SIRS-based definition, the reported incidence of sepsis increased dramatically, a change that was also driven by the pressure of in-hospital financial incentives [8]. However, the actual number of patients with sepsis, as defined using the criteria of infection plus organ dysfunction, did not increase substantially [9,10].

### 2.4. Third Sepsis Definitions Conference

There was therefore a pressing need to return to the concept that sepsis is a ‘bad infection’, i.e., an infection plus some organ failure attributed to it [11]; this was concretized in another consensus conference in 2016 (the so-called third one) organized by the SCCM and the European Society of Intensive Care Medicine (ESICM) [12]. This group also reiterated that septic shock includes the added criteria of refractory hypotension requiring vasopressor therapy and increased blood lactate levels, typically > 2 mmol/L [13]. To objectively assess the changes in organ function, the sequential organ function assessment (SOFA) score [14] was thought to be the most appropriate tool (Table 4), with the criterion being an increase of at least 2 points from baseline.

## 3. Conclusions

Sepsis is, and has always been, an infection associated with some degree of organ dysfunction. The clinical signs and symptoms of infection have not changed over time, and it was an error of the so-called SCCM/ACCP conference [5] to propose to use the same signs of infection for sepsis identification. One cannot underestimate the importance of early recognition and treatment of sepsis, and this creates a need for easy patient identification. The clinician has always thought of sepsis as a ‘bad infection’, i.e., an infection associated with some degree of acute alteration in organ function. When more objective criteria are needed, as in clinical trials, a SOFA score can be used to quantify the degree of organ dysfunction.

The early recognition of sepsis and its severity is important to enable appropriate treatment to be started rapidly to optimize the chances of survival. Mortality rates are still around 30–35% for sepsis and 50–60% for septic shock, but these values vary substantially across units and countries [11]. Importantly, these mortality rates usually refer to ICU patients, but many people likely die of sepsis outside the ICU, some of whom will develop sepsis at the end of their lives, whether that be due to terminal cancer, terminal organ failure, or simply old age [12]. In some of these patients, sepsis will not need any treatment except compassionate end-of-life care. With the pressure (from media, patients, and peers) for physicians to be ultra-aware of the risks of sepsis, one should be cautious about reflex treatment, what some experts called ‘sepsis hysteria’ [15], in these patients, and in patients who may not actually have sepsis. As our ability to identify sepsis improves using new biomarkers and ‘omics technology [16], definitions may evolve again to include some of these criteria; however, whichever definition is adopted, terminology must not get in the way of providing best possible patient care at the bedside.

## Figures and Tables

**Table 1:** Simplified presentation of some medical constructs.

Construct	Pathophysiology	Clinical Features
Sepsis	Dysregulated host response to infection	Infection + organ dysfunction
ARDS	Pulmonary edema due to leaky capillaries	Severe hypoxemia Bilateral lung infiltrates No evidence of hemodynamic type lung edema
Coma	Altered brain function	Altered consciousness
Dementia	Damage, degeneration, or loss of brain cells and/or their connections	Confusion Loss of memory Abnormal behavior

ARDS: acute respiratory distress syndrome.

**Table 2:** Comparison of definitions and criteria for two common ICU conditions.

Condition	Definition	Criteria
Myocardial infarction	Thrombotic event in a coronary artery	Abnormal EKG, elevated blood troponin levels
Pneumonia	Pulmonary infection	Fever, abnormal WBC count, raised CRP, abnormal chest X-ray/CT scan

WBC: white blood cell; CRP: C-reactive protein; CT: computed tomography; EKG: electrocardiogram.

**Table 3:** Signs of sepsis as listed at the 2001 Sepsis Definitions conference [7].

Category	Sign/Symptom
General	Rigor–fever (sometimes hypothermia) Tachypnea/respiratory alkalosis Positive fluid balance–edema
Hematologic/inflammatory reaction	Leukocytosis (sometimes leukopenia)–increased immature forms Increased CRP, IL-6, procalcitonin concentrations
Hemodynamic alterations	Arterial hypotension Tachycardia Increased cardiac output/low SVR/high SvO_2_ Altered skin perfusion (cold, mottled extremities, petechiae, etc.) Decreased urine output Hyperlactatemia–increased base deficit
Signs of organ dysfunction	Hypoxemia (ALI/ARDS) Altered mental status Altered renal function Hyperglycemia Thrombocytopenia–DIC Intolerance to feeding (altered gut motility) Altered liver tests (hyperbilirubinemia)

CRP: C-reactive protein; IL: interleukin; SVR: systemic vascular resistance; SvO_2_: mixed venous oxygen saturation; ALI: acute lung injury; ARDS: acute respiratory syndrome; DIC: disseminated intravascular coagulation.

**Table 4:** Sequential Organ Failure Assessment (SOFA) score [14].

Score	0	1	2	3	4
**Respiratory**					
PaO_2_/FiO_2_, mmHg	≥400	<400	<300	<200	<100
				--------with respiratory support-------	
**Coagulation**					
Platelets × 10^3^/mm^3^	≥150	<150	<100	<50	<20
**Liver**					
Bilirubin, mg/dL (μmol/L)	<1.2 (<20)	1.2–1.9 (20–32)	2.0–5.9 (33–101)	6.0–11.9 (102–204)	>12.0 (>204)
**Cardiovascular**					
Hypotension	No hypotension	MAP < 70 mmHg	dopamine ≤ 5 or dobutamine (any dose) *	dopamine > 5 or epinephrine ≤ 0.1 or norepinephrine ≤ 0.1 *	dopamine > 15 or epinephrine > 0.1 or norepinephrine > 0.1 *
**Central nervous system**					
Glasgow Coma Scale	15	13–14	10–12	6–9	<6
**Renal**					
Creatinine, mg/dL (μmol/L)	<1.2 (<110)	1.2–1.9 (110–170)	2.0–3.4 (171–299)	3.5–4.9 (300–440)	>5.0 (>440)
OR urine output				<500 mL/d	<200 mL/d

* adrenergic agents administered for at least one hour (doses given are in mcg/kg/min).

## Data Availability

Not applicable.
